# Trends in Utilization and Outcomes of Isolated and Concomitant Tricuspid Valve Surgery in the United States

**DOI:** 10.1016/j.atssr.2025.04.015

**Published:** 2025-05-15

**Authors:** Ayesha P. Ng, Joseph E. Hadaya, Esteban Aguayo, Konmal Ali, Troy N. Coaston, Peyman Benharash

**Affiliations:** 1 Cardiovascular Outcomes Research Laboratories, David Geffen School of Medicine at UCLA, Los Angeles, California; 2 Division of Cardiac Surgery, Department of Surgery, David Geffen School of Medicine at UCLA, Los Angeles, California

## Abstract

**Background:**

Despite the increasing prevalence of tricuspid valve (TV) regurgitation, surgical interventions remain low, potentially due to high operative mortality. Given the lack of contemporary data, we examined trends in utilization and outcomes after isolated and concomitant TV operations.

**Methods:**

Adults undergoing TV repair/replacement were identified in the 2016-2021 National Inpatient Sample. Patients undergoing heart transplantation, ventricular assist device placement, or with endocarditis were excluded. Study cohorts were stratified based on Isolated vs concomitant TV surgery (TV-Mitral, TV-Aortic, TV-coronary artery bypass grafting [CABG]). Multivariable mixed regressions were developed to evaluate the association of concomitant surgery with major adverse events including mortality and complications, costs, and length of stay.

**Results:**

Of 51,940 patients, 19.2% underwent Isolated TV, 47.2% TV-Mitral, 14.6% TV-Aortic, and 19.0% TV-CABG operations. The volume of Isolated TV procedures significantly increased from 1415 in 2016 to 1830 in 2021 (*P* = .001). Compared with Isolated TV, patients undergoing concomitant operations were older with greater burden of comorbidities. TV-CABG and TV-Aortic patients experienced higher major adverse event rates of 67.6% and 56.5%, respectively, compared with 46.1% and 50.1% among TV-Mitral and Isolated TV (*P* < .001). Furthermore, TV-CABG and TV-Aortic cohorts experienced greater length of stay, costs, and nonhome discharge relative to TV-Mitral and Isolated TV, which were comparable. After adjustment, major adverse event rates significantly decreased over time among TV-CABG and remained stable among all other groups.

**Conclusions:**

Utilization of isolated tricuspid surgery is rising, with comparable complications and resource use relative to concomitant mitral operations. Given the lack of improvement in postoperative morbidity over time, further optimization of tricuspid surgical timing is warranted.

**In Short**

▪ Utilization of isolated tricuspid valve surgery increased over time.
▪ Concomitant aortic and coronary artery bypass grafting operations had greater complications and resource use relative to concomitant mitral and isolated tricuspid operations, which were comparable.
▪ Given the lack of improvement in postoperative morbidity over time, further optimization of tricuspid surgical timing is warranted.

Severe tricuspid regurgitation (TR) affects over 1.6 million people in the United States and may lead to progressive right ventricular dilation and heart failure if left untreated.^1^ Although the prevalence of TR is increasing, surgical interventions remain underutilized.^1^ Historically, tricuspid valve (TV) operations have been associated with higher postoperative morbidity and mortality compared with mitral or aortic valve procedures.^2^^,^^3^

The optimal approach regarding the type and timing of intervention for TR remains controversial. Secondary or functional etiology is the most common cause of TR and thus the majority of TV procedures are performed concomitantly with other cardiac operations.^3^ Nevertheless, primary TR is growing due to causes such as atrial fibrillation and prior intracardiac device placement.^2^ While isolated TV procedures carry higher risk compared with isolated aortic or mitral valve operations, concomitant TV operations have been associated with the greatest risk of mortality.^3^ However, outcomes of isolated and concomitant TV operations have yet to be examined in the contemporary era.

Using a nationally representative cohort, we aimed to characterize trends in utilization and compare in-hospital outcomes after isolated and concomitant TV operations.

## Patients and Methods

### Study Population

This was a retrospective cohort study using the 2016-2021 National Inpatient Sample (NIS). Maintained by the Healthcare Cost and Utilization Project, the NIS provides accurate estimates for approximately 97% of all US hospitalizations.^4^ Due to the deidentified nature of the NIS, this study was deemed exempt from full review by the institutional review board at the University of California, Los Angeles.

All adults (≥18 years) undergoing TV repair or replacement were identified using relevant International Classification of Diseases, 10th Revision, procedure codes (Supplemental Table 1). Patients undergoing heart transplantation, ventricular assist device placement, or with history of endocarditis were excluded (Supplemental Table 1). Study cohorts were stratified based on whether the TV operation was isolated (Isolated TV) or performed concomitantly with another cardiac procedure (TV-Mitral, TV-Aortic, TV-coronary artery bypass grafting (CABG).

### Statistical Analysis

Categorical and continuous variables are reported as proportion (%) or median (interquartile range) and compared using the Pearson’s χ^2^ or Kruskal-Wallis tests, respectively. Significance of temporal trends was assessed using Cuzick’s nonparametric test. Multivariable linear and logistic regressions were developed to evaluate the association of concomitant TV surgery with outcomes of interest. Variable selection for all models was performed by applying the Least Absolute Shrinkage and Selection Operator algorithm.^5^ An interaction term was used to analyze differences in rates of major adverse events (MAEs), defined as a composite of mortality and complications, over time. Statistical significance was set at α = 0.05. All statistical analyses were performed using Stata 16.1 (StataCorp).

## Results

**Figure 1:**
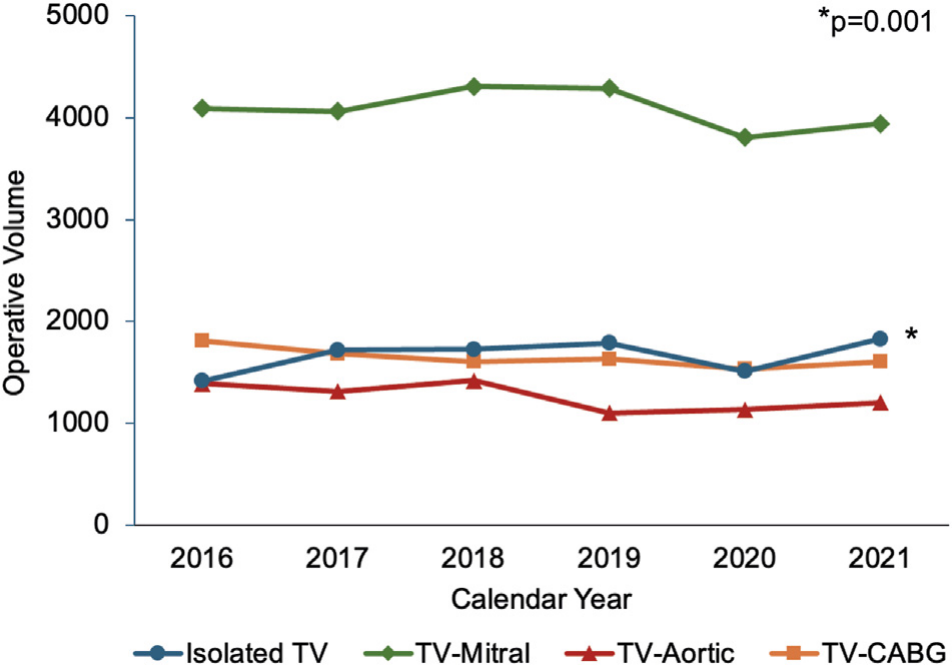
Temporal trends in utilization of isolated and concomitant tricuspid valve (TV) surgery. (CABG, coronary artery bypass grafting.)

Of 51,940 patients undergoing TV repair or replacement, 9990 (19.2%) underwent Isolated TV, 24,515 (47.2%) underwent TV-Mitral, 7565 (14.6%) underwent TV-Aortic, and 9870 (19.0%) underwent TV-CABG operations. Over the study period, Isolated TV procedures significantly increased from 1415 cases in 2016 to 1830 in 2021 (*P* = .001), while the volume of all concomitant operations remained stable (Figure 1).

Compared with Isolated TV, patients undergoing concomitant operations were older, with greater burden of comorbidities, and more commonly publicly insured (Table 1). While the rate of prior CABG was comparable between groups, Isolated TV and TV-Aortic patients had significantly greater rates of prior heart valve surgery compared with TV-Mitral and TV-CABG (4.4% vs 2.8% vs 1.7% vs 1.1%, *P* < .001). In addition, the TV-Mitral group had the highest rate of concomitant maze procedures (32.4% vs 15.4% vs 19.3% vs 22.1%, *P* < .001), and Isolated TV had the lowest rate of valve repair rather than replacement (50.9% vs 94.2% vs 91.6% vs 93.5%, *P* < .001, Table 1).

**Table 1:** Patient and Hospital Characteristics Stratified by Isolated or Concomitant Tricuspid Valve Surgery

Parameter	Isolated TV (n = 9990)	TV-Mitral (n = 24,515)	TV-Aortic (n = 7565)	TV-CABG (n = 9870)	*P* Value
Age, y	57 (38-70)	68 (59-75)	69 (58-76)	71 (63-77)	<.001
Female sex	59.0	59.3	52.8	40.4	<.001
Race					.001
White	72.4	72.5	70.4	74.3	
Black	11.1	12.8	12.7	10.6	
Hispanic	9.2	6.9	8.0	7.3	
Asian	4.5	4.3	4.0	3.4	
Other	2.8	3.5	4.8	4.3	
Payer Status					<.001
Private	31.4	26.5	22.8	16.9	
Medicare	41.6	60.8	63.2	72.0	
Medicaid	21.4	8.8	10.2	7.1	
Other	5.6	3.9	3.7	4.0	
Comorbidities					
Elixhauser Comorbidity Index (median, IQR)	5 (4-6)	6 (4-7)	6 (5-7)	6 (4-7)	<.001
Congestive heart failure	59.9	70.3	75.2	76.8	<.001
Coronary artery disease	25.2	33.6	37.0	82.8	<.001
Hypertension	59.9	73.8	76.0	82.2	<.001
Atrial fibrillation	51.4	77.6	70.6	68.8	<.001
Pulmonary circulatory disorder	32.4	46.0	42.6	39.2	<.001
Chronic lung disease	19.9	22.7	24.3	24.5	.002
Chronic liver disease	13.7	6.3	9.8	9.5	<.001
Chronic kidney disease	4.8	4.2	7.0	10.0	<.001
Diabetes	17.0	19.4	22.2	36.4	<.001
Peripheral vascular disease	11.6	10.6	22.9	18.1	<.001
Coagulopathy	39.9	43.0	54.5	47.1	<.001
Nonrheumatic tricuspid regurgitation	33.1	24.0	20.9	21.5	<.001
Prior procedure					
Presence of pacemaker	6.2	4.7	4.6	3.2	<.001
Prior valve surgery	4.4	1.7	2.8	1.1	<.001
Prior CABG	1.9	1.4	1.1	1.9	.15
Nonelective admission	29.0	25.9	31.7	41.3	<.001
Robot-assisted	0.9	1.5	0.1	0.1	<.001
Concomitant maze	15.4	32.4	19.3	22.1	<.001
Operative type					
TV repair	50.9	94.2	91.6	93.5	<.001
TV replacement (bioprosthetic)	41.4	4.5	7.1	5.2	<.001
TV replacement (mechanical)	7.7	1.2	1.3	1.3	<.001
Mitral/aortic valve repair	…	47.5	4.1	…	
Mitral/aortic valve replacement	…	52.5	95.9	…	
*Hospital volume tertile*					<.001
Low	7.8	8.8	7.3	10.2	
Medium	27.6	29.8	26.4	33.7	
High	64.6	61.5	66.3	56.1	
*Hospital region*					<.001
South	31.3	31.2	26.6	30.0	
Northeast	17.3	23.8	22.3	19.3	
Midwest	31.8	27.3	31.1	31.8	
West	19.6	17.7	20.0	18.9	
*Hospital teaching status*					<.001
Nonmetropolitan	0.7	1.2	1.0	1.9	
Metropolitan nonteaching	5.4	8.1	7.7	9.0	
Metropolitan teaching	93.9	90.7	91.3	89.1	

Values are presented as percent or median (interquartile range (IQR)).

CABG, coronary artery bypass grafting; TV, tricuspid valve.

Concomitant TV-CABG and TV-Aortic patients experienced higher mortality rates of 10.4% and 8.3%, respectively, compared with 3.7% and 4.7% among TV-Mitral and Isolated TV patients (*P* < .001). Furthermore, the TV-Mitral group demonstrated the lowest rate of complications among all groups (Table 2). Patients undergoing TV-CABG had the lowest rate of pacemaker implantation at 7.2%, while all other groups were similar at 10% (*P* < .001). The TV-CABG and TV-Aortic cohorts experienced greater LOS, costs, and non-home discharge relative to TV-Mitral and Isolated TV, which were comparable (Table 2).

**Table 2:** Unadjusted Outcomes Stratified by Isolated or Concomitant Tricuspid Valve Surgery

Outcome	Isolated TV (n = 9990)	TV-Mitral (n = 24,515)	TV-Aortic (n = 7565)	TV-CABG (n = 9870)	*P* Value
In-hospital mortality	4.7	3.7	8.3	10.4	<.001
Complications					
Cardiac	19.3	19.9	24.1	39.2	<.001
Respiratory	16.6	15.0	21.2	23.7	<.001
Renal	26.3	25.9	34.6	39.8	<.001
Infectious	9.3	4.4	7.1	7.4	<.001
Thromboembolic	5.6	0.8	1.4	1.1	<.001
Cerebrovascular	0.5	0.6	1.8	0.7	<.001
Hemorrhagic	4.1	3.7	5.1	4.7	.07
MAE	50.1	46.1	56.5	67.6	<.001
Blood transfusion	20.6	24.6	27.4	26.4	<.001
Permanent pacemaker implantation	10.4	10.9	10.8	7.2	<.001
LOS, d	9 (6-16)	9 (6-14)	11 (8-18)	12 (8-19)	<.001
Cost, $1000s	59.8 (42.3-95.5)	60.8 (45.4-86.5)	77.9 (58.2-115.1)	76.9 (55.7-112.9)	<.001
Nonhome discharge	24.3	26.7	37.9	47.4	<.001

Values are presented as percent or median (interquartile range). Major adverse event (MAE) was defined as a composite of in-hospital mortality and complications.

CABG, coronary artery bypass grafting; LOS, length of stay; TV, tricuspid valve.

**Figure 2:**
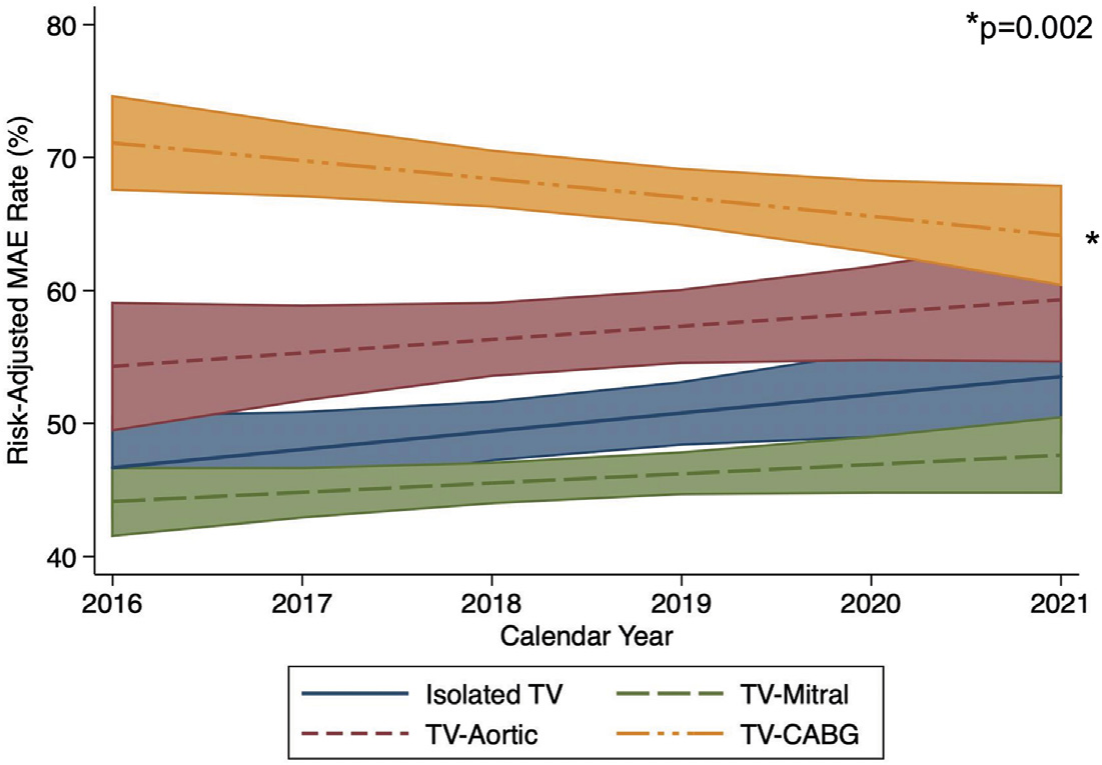
Temporal trends in risk-adjusted rates of major adverse event (MAE), defined as a composite of in-hospital mortality and complications, stratified by isolated or concomitant tricuspid valve (TV) surgery. (CABG, Coronary artery bypass grafting.)

Following adjustment, TV-Mitral was associated with significantly lower odds of MAE relative to Isolated TV (adjusted odds ratio [AOR], 0.73; 95% CI, 0.65-0.83), while TV-CABG exhibited the opposite association (AOR, 1.45; 95% CI, 1.25-1.68; Supplemental Table 2). Although risk-adjusted rates of MAE significantly decreased among the TV-CABG group over time (*P* = .002), MAE rates remained stable among all other groups (Figure 2). In addition, high-volume hospitals were associated with significantly lower odds of MAE compared with low-volume centers (AOR, 0.83; 95% CI, 0.70-0.97).

Concomitant TV-Mitral operations were associated with a –1.8 day decrement in length of stay (LOS) (95% CI, –2.5 to –.1) and –$10,100 decrease in episodic costs (95% CI, –$14,900 to –$5200) relative to Isolated TV, while the TV-Aortic and TV-CABG groups demonstrated notably increased costs (Supplemental Table 3). Additionally, patients with TV-Mitral operations experienced lower odds of nonhome discharge (AOR, 0.71; 95% CI, 0.61-0.82) compared with Isolated TV.

On sensitivity analysis among patients with a primary diagnosis of TR, TV-Mitral patients demonstrated comparable rates of mortality (5.3 vs 4.2%, *P* = .07), MAE (47.4 vs 39.5%, *P* = .29), and pacemaker implantation (6.5 vs 11.9%, *P* = .18) compared with Isolated TV. Although TV-Mitral patients had increased LOS (10 vs 8 days, *P* < .001) and costs ($66,900 vs 51,400, *P* < .001) on unadjusted analysis, both LOS and costs were comparable after adjustment for patient, operative, and hospital factors (Supplemental Table 4).

## Comment

Despite the increasing utilization of isolated TV surgery, postoperative morbidity remained unchanged over the study period. Notably, patients undergoing concomitant TV-Aortic and TV-CABG operations experienced considerably greater mortality, complications, LOS, and costs relative to concomitant TV-mitral and isolated TV operations, which were comparable.

Consistent with literature from The Society of Thoracic Surgeons Adult Cardiac Surgery Database, we demonstrated approximately 2000 isolated TV operations per year.^6^ We reported a mortality rate of 5% after isolated TV operations, which remains relatively high compared with isolated aortic or mitral valve surgery, with rates of 2% to 3%.^2^ According to the current American College of Cardiology/American Heart Association (ACC/AHA) guidelines, isolated TV surgery is a class IIa recommendation for symptomatic patients with severe primary or secondary TR.^7^ However, patients have historically not been referred for isolated TV surgery until late in the course of disease. Growing interest in earlier operation for severe isolated TR before the onset of right ventricular dysfunction and end-organ damage may help lower operative risk.^8^

In contrast with delayed intervention for isolated TR, mitral surgery is often performed prior to the onset of left ventricular dysfunction or symptoms, which may underlie the improved outcomes of concomitant TV-Mitral operations.^8^ For patients undergoing left-sided valve surgery with severe TR, concomitant TV surgery is a class I recommendation in the ACC/AHA guidelines.^7^ For mild to moderate TR, some argue that mitral surgery will restore tricuspid valve function and may refrain from concomitant TV repair. However, untreated secondary TR can progress, and reoperation for severe, isolated TR after mitral surgery is associated with a steep mortality rate of 10% to 25%.^7^^,^^8^ Moreover, intervention for TR generally does not add to the risks of mitral valve surgery and has been shown to have long-term efficacy.^9^ Our findings suggest that concomitant TV-Mitral operations are safe and when appropriate should not be deferred. Further efforts to optimize patient selection criteria and timing of intervention for primary and secondary TR are warranted. Serial assessments for progression of TR, including TV annular dilation and right ventricular size and function, along with refinement of surgical risk scores, may help guide clinical decision-making.^10^

This study has several limitations inherent to its retrospective nature and use of administrative data. The NIS lacks clinical granularity regarding information such as the primary indication for surgery, TR severity, right ventricular size and function, and Society of Thoracic Surgeons score. All available data were limited to the duration of hospitalization and did not capture outpatient data, readmissions, or long-term outcomes. International Classification of Diseases coding may be influenced by provider and hospital practices among participating centers in the NIS. Nevertheless, we used the largest all-payer inpatient database and robust statistical methods to enhance the generalizability of our findings at the national level.

In conclusion, the utilization of isolated TV surgery is rising. Notably, isolated TV and concomitant TV-mitral operations demonstrated similarly low mortality, complications, LOS, and costs compared with other concomitant TV procedures. Our findings suggest that concomitant TV-mitral surgery is safe and cost-effective, and, when appropriate, should not be deferred. Given the lack of improvement in postoperative morbidity over time, further optimization of surgical timing particularly for isolated TV surgery is warranted.

## References

[1] Axtell A.L., Bhambhani V., Moonsamy P. (2019). Surgery does not improve survival in patients with isolated severe tricuspid regurgitation. J Am Coll Cardiol.

[2] Zack C.J., Fender E.A., Chandrashekar P. (2017). National trends and outcomes in isolated tricuspid valve surgery. J Am Coll Cardiol.

[3] Vassileva C.M., Shabosky J., Boley T., Markwell S., Hazelrigg S. (2012). Tricuspid valve surgery: the past 10 years from the Nationwide Inpatient Sample (NIS) database. J Thor Cardiovasc Surg.

[4] HCUP National Inpatient Sample (NIS) (2021). Healthcare Cost and Utilization Project (HCUP), Agency for Healthcare Research and Quality. http://www.hcup-us.ahrq.gov/nisoverview.jsp.

[5] Tibshirani R. (1996). Regression shrinkage and selection via the lasso. J R Stat Soc Ser B.

[6] Chen Q., Bowdish M.E., Malas J. (2023). Isolated tricuspid operations: The Society of Thoracic Surgeons Adult Cardiac Surgery Database analysis. Ann Thorac Surg.

[7] Otto C.M., Nishimura R.A., Bonow R.O. (2021). 2020 ACC/AHA Guideline for the Management of Patients With Valvular Heart Disease: executive summary: a report of the American College of Cardiology/American Heart Association Joint Committee on Clinical Practice Guidelines. J Am Coll Cardiol.

[8] Gammie J.S., Chu M.W., Falk V. (2022). Concomitant tricuspid repair in patients with degenerative mitral regurgitation. New Engl J Med.

[9] Cetinkaya A., Ganchewa N., Hein S. (2020). Long-term outcomes of concomitant tricuspid valve repair in patients undergoing mitral valve surgery. J Cardiothorac Surg.

[10] Dreyfus J., Audureau E., Bohbot Y. (2022). TRI-SCORE: a new risk score for in-hospital mortality prediction after isolated tricuspid valve surgery. Eur Heart J.

