# Supplementary material for: Trends in Utilization and Outcomes of Isolated and Concomitant Tricuspid Valve Surgery in the United States
Source: Ann Thorac Surg Short Rep. 2025 May 15;3(4):1017–22. doi: 10.1016/j.atssr.2025.04.015 (PMC12712209; doi:10.1016/j.atssr.2025.04.015)
Supplement: Supplementary Tables 1-4 [file mmc1.docx]

Supplemental Table 1. Administrative *International Classification of Diseases, 10^th^ Revision* (ICD-10) diagnosis and procedure codes for tricuspid valve surgery.

|  | **ICD-10** |
| --- | --- |
| **Cardiac Operations** |  |
| Tricuspid Valve Replacement | 02RJ07x, 02RJ08x, 02RJ0Jx, 02RJ0Kx  02RJ47x, 02RJ48x, 02RJ4Jx, 02RJ4Kx |
| Mechanical Tricuspid Valve Replacement | 02RJ0J, 02RJ4J |
| Bioprosthetic Tricuspid Valve Replacement | 02RJ07, 02RJ08, 02RJ0K, 02RJ47, 02RJ48, 02RJ4K |
| Tricuspid Valve Repair | 02QJ0Z, 02QJ4Z |
| Aortic Valve Replacement | 02RF07x, 02RF08x, 02RF0Jx, 02RF0Kx, 02RF47x, 02RF48x, 02RF4Jx, 02RF4Kx, |
| Aortic Valve Repair | 02QF0Zx 02QF4Zx, 02UF07x, 02UF08x, 02UF0Jx, 02UF0K, 02UF47x, 02UF48x, 02UF4Jx, 02UF4Kx |
| Mitral Valve Replacement | 02RG07x, 02RG08x, 02RG0Jx, 02RG0Kx  02RG47x, 02RG48x, 02RG4Jx, 02RG4Kx |
| Mitral Valve Repair | 02QG0Z, 02QG4Z,  02UG07x, 02UG08x, 02UG0Jx, 02UG0Kx  02UG47x, 02UG48x, 02UG4Jx, 02UG4Kx |
| Coronary artery bypass graft (CABG) | 02100x, 02110x, 02120x, 02130x |
| Robot-Assisted | 8E0W4CZ, 8E0W8CZ |
| Heart Transplantation | 02YA0Z0 |
| Durable Ventricular Assist Device Placement | 02HA0QZ, 02WA0QZ, 02WA3QZ, 02WA4QZ |
| Maze procedure | 02560ZZ, 02563ZZ, 02564ZZ, 02570ZZ, 02573ZZ, 02574ZZ, 02580ZZ, 02583ZZ, 02584ZZ, 02B60ZZ, 02B63ZZ, 02B64ZZ, 02B70ZZ, 02B73ZZ, 02B74ZZ, 02B80ZZ, 02B83ZZ, 02B84ZZ |
|  |  |
| **Comorbidities** |  |
| Endocarditis | I33, I38, I39 |
| Congestive Heart Failure | I43, I50, I09.9, I11.0, I13.0, I25.5, I42.0, I42.5, I42.6, I42.7, I42.8, I42.9, P29.0 |
| Coronary Artery Disease | I20, I25 |
| Hypertension | I10, I11, I12, I13, I15 |
| Atrial fibrillation | I48 |
| Pulmonary Circulatory Disorder | I26, I27, I28 |
| Chronic Lung Disease | I27.8, I27.9, J68.4, J70.1, J70.3, J40, J41, J42, J43, J44, J45, J46, J47, J60, J61, J62, J63, J64, J65, J66, J67 |
| Chronic Liver Disease | K70, K72, K73, K74, B18, I85, K76.3, K76.4, K76.5, K76.6, K76.7, K76.8, K76.9, Z94.4, K71.1, K71.3, K71.4, K71.5, K71.7, K76.0, K76.2, I86.4, I98.2 |
| Chronic Kidney Disease | Z49, N19, I12.0, N25.0, Z94.0, N18.5, N18.6, I13.11, V45.1, Z99.2, Z91.15 |
| Diabetes | E10, E11, E13 |
| Peripheral Vascular Disease | I70, I71, I73, I77 |
| Coagulopathy | D65, D66, D67, D68, D69 |
| Tricuspid regurgitation | I36.1 |
| Mitral regurgitation | I34.0 |
| Presence of pacemaker | Z95.0, Z95.810 |
| Prior valve surgery | Z95.2, Z95.3, Z95.4 |
| Prior CABG | Z95.1 |
|  |  |
| **Complications** |  |
| Stroke | I63, I67.2, I67.81, I67.82, I67.89, I67.7, I67.5, I67.9, G97.81, G97.82, I97.811, I97.821, I61.9, I62.1, I60.9, I62.9, I62.0, I62.1 |
| Deep Vein Thrombosis | I82.220, I82.4, I82.6, I82.A1, I82.B1, I82.C1, I82.290, I82.890, I82.91, I80.9, I80.3 |
| Pulmonary Embolism | I26 |
| Respiratory Failure | J96.00, J96.90, J96.20, J95.821, J95.822 |
| Prolonged Mechanical Ventilation | 5A1955Z |
| Pneumonia | J12, J13, J14, J15, J16, J18, J95.851, J95.89 |
| Sepsis | A40, A41, R65.20, T814XXA, K68.11 |
| Wound infection | T81.32XA, T81.31XA, T81.4XXA, K68.11 |
| Acute Kidney Injury | N17 |
| Cardiac Arrest | I46.2, I46.8, I46.9 |
| Myocardial Infarction | I21, I22, I23 |
| Cardiac tamponade | I31.4 |
| Hemorrhage | E36.01, E36.02, E89.810, E89.811, G97.31, G97.32, G97.51, G97.52, H59.111, H59.112, H59.113, H59.119, H59.121, H59.122, H59.123, H59.129, H59.311, H59.312, H59.313, H59.319, H59.321, H59.322, H59.323, H59.329, H95.21, H95.22, H95.41, H95.42, I97.410, I97.411, I97.418, I97.42, I97.610, I97.611, I97.618, I96.620, J95.61, J95.62, J95.830, J95.831, K91.61, K91.62, K91.840, K91.841, L76.01, L76.02, L76.21, L76.22, M96.810, M96.811, M96.830, M96.831, N99.61, N99.62, N99.820, N99.821 |
| Blood transfusion | 30233H0, 30233N0, 30243H0, 30243N0, 30253H0, 30253N0, 30263H0, 30263N0, 30233H1, 30243H1, 30253H1, 30263H1, 30233H0, 30233N0, 30233W0, 30243H0, 30243N0, 30243W0, 30253H0, 30253N0, 30253W0, 30233H0, 30233N0, 30233W0, 30243H0, 30263H0, 30263N0, 30263W0, 30233H1, 30243H1, 30253H1, 30263H1, 30233N1, 30233P1, 30243N1, 30243P1, 30253N1, 30253P1, 30263N1, 30263P1, 30233R1, 30243R1, 30253R1, 30263R1, 30233T1, 30233V1, 30233W1, 30243T1, 30243V1, 30243W1, 30253T1, 30253V1, 30253W1, 30263T1, 30263V1, 30263W1, 30233J1, 30233K1, 30233L1, 30233M1, 30243J1, 30243K1, 30243L1, 30243M1, 30253J1, 30253K1, 30253L1, 30253M1, 30263J1, 30263K1, 30263L1, 30263M1, 3E033GC, 3E043GC, 3E053GC, 3E063GC, 30233Q1, 30243Q1, 30253Q1, 30263Q1 |
| Pacemaker implantation | 0JH60PZ, 0JH63PZ, 0JH604Z, 0JH634Z, 0JH605Z, 0JH635Z, 0JH606Z, 0JH636Z |
|  |  |

Supplemental Table 2. Factors associated with major adverse events, defined as a composite of in-hospital mortality and complications, among patients undergoing tricuspid valve (TV) surgery. Model C-statistic: 0.70. *Ref: Reference. AOR: Adjusted odds ratio. CI: Confidence interval.*

| **Parameter** | **AOR [95% CI]** | **p-value** |
| --- | --- | --- |
| Age (per year) | 1.01 [1.00-1.01] | <0.001 |
| Female sex (ref: male) | 0.83 [0.77-0.91] | <0.001 |
| *Race* |  |  |
| White | Ref |  |
| Black | 1.31 [1.14-1.51] | <0.001 |
| Hispanic | 1.08 [0.92-1.28] | 0.34 |
| Asian | 1.03 [0.83-1.28] | 0.82 |
| Other | 0.91 [0.71-1.15] | 0.42 |
| *Payer Status* |  |  |
| Private | Ref |  |
| Medicare | 1.19 [1.05-1.35] | 0.004 |
| Medicaid | 1.35 [1.15-1.59] | <0.001 |
| Other | 1.48 [1.17-1.86] | 0.001 |
| *Comorbidities* |  |  |
| Elixhauser Comorbidity Index | 0.98 [0.95-1.01] | 0.34 |
| Congestive heart failure | 1.78 [1.61-1.97] | <0.001 |
| Chronic liver disease | 2.51 [2.12-2.98] | <0.001 |
| Tricuspid regurgitation | 0.79 [0.70-0.88] | <0.001 |
| *Prior procedure* |  |  |
| Presence of pacemaker | 0.69 [0.56-0.86] | 0.001 |
| Prior valve surgery | 0.63 [0.47-0.84] | 0.002 |
| Prior CABG | 0.69 [0.48-1.00] | 0.05 |
| Non-elective admission | 2.68 [2.41-2.98] | <0.001 |
| Robot-assisted | 0.71 [0.42-1.20] | 0.20 |
| *Type of operation* |  |  |
| Isolated TV | Ref |  |
| TV-Mitral | 0.73 [0.65-0.83] | <0.001 |
| TV-Aortic | 1.04 [0.88-1.23] | 0.62 |
| TV-CABG | 1.45 [1.25-1.68] | <0.001 |
| *Hospital Volume Tertile* |  |  |
| Low | Ref |  |
| Medium | 0.87 [0.74-1.03] | 0.11 |
| High | 0.83 [0.70-0.97] | 0.03 |
| *Hospital Region* |  |  |
| South | Ref |  |
| Northeast | 0.87 [0.76-0.99] | 0.04 |
| Midwest | 0.80 [0.69-0.93] | 0.004 |
| West | 1.00 [0.88-1.14] | 0.99 |
| *Hospital Teaching Status* |  |  |
| Non-metropolitan | Ref |  |
| Metropolitan non-teaching | 1.31 [0.84-2.05] | 0.23 |
| Metropolitan teaching | 1.31 [0.86-2.00] | 0.20 |

Supplemental Table 3. Adjusted outcomes associated with concomitant tricuspid valve (TV) surgery with reference to isolated TV surgery. *Major adverse events (MAE) were defined as a composite of in-hospital mortality and complications.* *Ref: Reference. AOR: Adjusted odds ratio. ß: Beta coefficient. CI: Confidence interval. LOS: Length of stay. Isolated TV: Isolated tricuspid operations. TV-Mitral: Concomitant tricuspid-mitral operations. TV-Aortic: Concomitant tricuspid-aortic operations. TV-CABG: Concomitant tricuspid operations with coronary artery bypass graft.*

| **Outcome** | **Type of Operation** | **AOR or ß [95% CI]** | **p-value** |
| --- | --- | --- | --- |
| MAE | Isolated TV | Ref |  |
|  | TV-Mitral | 0.73 [0.65-0.83] | <0.001 |
|  | TV-Aortic | 1.04 [0.88-1.23] | 0.62 |
|  | TV-CABG | 1.45 [1.25-1.68] | <0.001 |
| LOS (days) | Isolated TV | Ref |  |
|  | TV-Mitral | -1.8 [-2.5, -1.1] | <0.001 |
|  | TV-Aortic | -0.1 [-0.9-0.8] | 0.92 |
|  | TV-CABG | +0.2 [-0.7-1.1] | 0.72 |
| Cost ($1000s) | Isolated TV | Ref |  |
|  | TV-Mitral | -10.1 [-14.9, -5.2] | <0.001 |
|  | TV-Aortic | +8.3 [1.8-14.7] | 0.01 |
|  | TV-CABG | +7.7 [1.4-14.1] | 0.02 |
| Non-home discharge | Isolated TV | Ref |  |
|  | TV-Mitral | 0.71 [0.61-0.82] | <0.001 |
|  | TV-Aortic | 1.19 [1.00-1.40] | 0.05 |
|  | TV-CABG | 1.43 [1.21-1.69] | <0.001 |

Supplemental Table 4. Sensitivity analysis comparing outcomes following isolated tricuspid valve (TV) surgery and concomitant tricuspid-mitral surgery among patients with primary diagnosis of tricuspid regurgitation. *Major adverse events (MAE) were defined as a composite of in-hospital mortality and complications.* *LOS: Length of stay. IQR: Interquartile range. CABG: Coronary artery bypass graft. ß: Beta-coefficient. CI: Confidence interval.*

| **Outcome** | **Isolated TV (n=2,355)** | **TV-Mitral**  **(n=310)** | **p-value** | **AOR / ß** | **95% CI** |
| --- | --- | --- | --- | --- | --- |
| In-hospital mortality (%) | 4.2 | 5.3 | 0.07 | 0.82 | 0.22-3.05 |
| *Complications (%)* |  |  |  |  |  |
| Cardiac | 14.6 | 24.6 | 0.25 | - | - |
| Respiratory | 12.7 | 5.3 | 0.07 | - | - |
| Renal | 23.6 | 31.6 | 0.18 | - | - |
| Infectious | 3.6 | 7.0 | 0.14 | - | - |
| Thromboembolic | 1.3 | 0 | 0.51 | - | - |
| Cerebrovascular | 0.8 | 0 | 0.83 | - | - |
| Hemorrhagic | 4.0 | 0 | 0.003 | - | - |
| MAE (%) | 39.5 | 47.4 | 0.29 | 1.06 | 0.56-2.03 |
| Blood transfusion (%) | 21.0 | 19.3 | 0.92 | 0.99 | 0.45-2.18 |
| Permanent pacemaker implantation (%) | 11.9 | 6.5 | 0.18 | 0.70 | 0.20-2.44 |
| LOS (days, median, IQR) | 8 [5-13] | 10 [6-17] | <0.001 | 0.9 | -2.4-4.2 |
| Cost ($1000s, median, IQR) | 51.4  [39.3-79.7] | 66.9  [50.9-92.6] | <0.001 | -1.2 | -8.6-16.2 |
| Non-home discharge (%) | 22.3 | 17.5 | 0.42 | 0.37 | 0.16-0.87 |
